# Artificial Intelligence Technology in Basketball Training Action Recognition

**DOI:** 10.3389/fnbot.2022.819784

**Published:** 2022-06-27

**Authors:** Yao Cheng, Xiaojun Liang, Yi Xu, Xin Kuang

**Affiliations:** ^1^Shaoxing University Yuanpei College, Shaoxing, China; ^2^College of Humanities, Zhaoqing Medical College, Zhaoqing, China; ^3^Graduate School, University of Perpetual Help System DALTA, Manila, Philippines; ^4^Ministry of Basic Education, Guangdong Eco-Engineering Polytechnic, Guangzhou, China; ^5^School of Management, Guang Dong AIB Polytechnic, Guangzhou, China

**Keywords:** somatosensory motion gesture recognition, recall, accuracy, artificial neural network, basketball training

## Abstract

The primary research purpose lies in studying the intelligent detection of movements in basketball training through artificial intelligence (AI) technology. Primarily, the theory of somatosensory gesture recognition is analyzed, which lays a theoretical foundation for research. Then, the collected signal is denoised and normalized to ensure that the obtained signal data will not be distorted. Finally, the four algorithms, decision tree (DT), naive Bayes (NB), support vector machine (SVM), and artificial neural network (ANN), are used to detect the data of athletes' different limb movements and recall. The accuracy of the data is compared and analyzed. Experiments show that the back propagation (BP) ANN algorithm has the best action recognition effect among the four algorithms. In basketball training athletes' upper limb movement detection, the average accuracy rate is close to 93.3%, and the average recall is also immediate to 93.3%. In basketball training athletes' lower limb movement detection, the average accuracy rate is close to 99.4%, and the average recall is immediate to 99.4%. In the detection of movements of upper and lower limbs: the recognition method can efficiently recognize the basketball actions of catching, passing, dribbling, and shooting, the recognition rate is over 95%, and the average accuracy of the four training actions of catching, passing, dribbling, and shooting is close to 98.95%. The intelligent basketball training system studied will help basketball coaches grasp the skilled movements of athletes better to make more efficient training programs and help athletes improve their skill level.

## Introduction

The rapid development of artificial intelligence (AI) technology in computer vision, mobile internet, big data analysis and other fields, deep learning, cross-border integration, and other technologies along with new features have gradually become the new focus of international competition. Additionally, in computer vision, AI technology combines cameras and computers to replace human eyes to segment, classify, and identify targets. These functions are used in virtual reality and human–computer interaction, especially in sports video analysis, which has become a research hotspot in industries and academia. Basketball is a collective sport. Compared with other sports, basketball is very technical. In basketball, the basketball level of players has a significant impact on the team (Gonzalo-Skok et al., 2019). Currently, basketball is very popular on college campuses. The development of basketball teaching is popularized. Many schools have created the conditions for education in basketball lessons. College students are also very keen to participate in basketball, and playing basketball has become the first choice for most students to enrich their extracurricular activities. However, the public basketball teaching and training in colleges and universities emphasize more on offense technique while ignoring defensive practice. If students can have a tactical training robot, it will significantly improve their basketball ability and technical and tactical level (Hagiwara et al., 2020). The analysis of sports technology can compare and evaluate athletes' training and game videos. After the technique is analyzed, the athlete's movement standardization, physical fitness, and other aspects are targeted for training. Additionally, in the team competition, the movement and position of the athletes are detected, tracked, and analyzed, which can promote the improvement of the technical level (Garcia et al., 2020). Basic movements in basketball games include dribbling, shooting, and layup. Dribbling is the most basic action in basketball, and shooting is the key to scoring the whole game. The accuracy of the basic movements has a greater impact on the game's score. With the development of basketball competitions, the human pose estimation algorithm is integrated with the action recognition algorithm. These algorithms play a vital role in assisting to improve the scoring rate. Human pose estimation detects and estimates the position, orientation, and scale information of each target human body part from the image. This information needs to be converted into a digital form that the computer can interpret and output the current human pose. However, action recognition is to judge whether a person's actions are normative and how to improve the normativeness according to pose estimation as the input object (Sobko et al., 2021).

This study explores the intelligent detection of movements in basketball training through AI technology. Firstly, relevant literature is researched and learned. The relevant theory of somatosensory gesture recognition is mastered. Secondly, the collected motion data are denoised and normalized. Finally, the superiority of the four algorithms is analyzed in terms of the recall rate and accuracy rate. The most suitable motion recognition algorithm is selected for the basketball training intelligent system. The experimental results may be applied to the research of basketball assistant robots. By basketball teaching assistant robots, basketball coaches can better grasp the technical movements of athletes, formulate more efficient training programs, and help athletes improve their technical level. The innovation point is to achieve more efficient detection of basketball players' technical movements during training through the four action recognition algorithms. By analyzing the recall rate and accuracy rate of the four algorithms, the optimal algorithm is selected to detect technical movements. These several rounds can serve as a good reference for developing basketball assistant robots in the future.

## Literature Review

### Related Research Analysis

With the development of AI technology pair, the technology is gradually applied in various fields. Several research teams have begun to study how to improve the quality of basketball training through AI technology.

Xu and Tang (2021) applied machine learning algorithms to the path planning of intelligent robots. Firstly, the movements in basketball training are identified and analyzed in combination with the basketball movement trajectory model. Secondly, a mathematical model of the trajectory of the basketball shot is established. An improved machine-learning based Q-learning algorithm is proposed. The algorithm realizes the path planning of the mobile robot and effectively completes the behavior of avoiding obstacles. The results show that the fuzzy controller applied to the basketball robot can effectively avoid the obstacles during the robot's motion. Therefore, the proposed machine learning algorithm has a good obstacle avoidance effect when applied to path planning in basketball training. The application of this algorithm can effectively prevent sports injuries in basketball (Xu and Tang, 2021). Zhi and Jiang (2020) proposed a path planning algorithm based on behavioral module control, aiming at problems, such as the strong dependence of traditional algorithms on environmental information, the path planning of basketball robots in unknown environments, and the improvement of autonomous navigation safety. They applied the fuzzy control theory to behavior control structures and combined these two path planning algorithms to solve the path planning problem of basketball robots in unknown environments. The results show that the basketball robot can overcome the uncertainty in the environment and effectively achieve good path planning, which verifies the feasibility of the fuzzy control algorithm and the validity and correctness of the path planning strategy (Zhi and Jiang, 2020). Cox et al. (2021) built a generic controller for regulating the motion of an inertia-driven jumping robot. The robot can specify the desired speed and jump height. The controller can ensure that the desired value is achieved. The controller can achieve the maximum response of the basketball robot the maximum jump height of 0.25 m (Cox et al., 2021). Carnevale et al. (2021) used the emerging distributed constrained aggregation optimization framework for application to basketball robots.

They proposed a constant-step distributed algorithm for solving online optimization problems. In the static case (i.e., with constant costs and constraints), they show that the knowledge estimate converges to the optimal solution at a linear rate. Finally, numerical values show that the method is effective in robotic basketball games and robotic surveillance scenarios (Carnevale et al., 2021). Yang (2020) expounded the development trend of AI technology, analyzed the development status of AI, and expounded the status of AI in sports. Finally, he focused on the AI training strategy for basketball players. Yang analyzed and studied the application in other aspects, aiming to provide theoretical support and guidance for further development of modern basketball. The basketball player training system should be based on AI, fully understand and evaluate the physical condition and competitive ability of the players, and measure the sports skills of the basketball players in time. The training system should propose training strategies according to the sports conditions of basketball players and optimize the training plan of the players (Yang, 2020).

When the action recognition technology based on AI technology is applied to the action recognition of basketball players, the action point data of the hand posture during the shooting process and the lower limb state during the movement process are collected. Basketball movements are complex movements completed by the upper and lower limbs. The identification of basic basketball movements plays an important role in improving the skills of basketball players. Therefore, the basic movements of the upper and lower limbs in basketball have been studied, and the identification of the basic movements of basketball has been preliminarily realized.

### Somatosensory Motion Gesture Recognition

**Figure 1:**
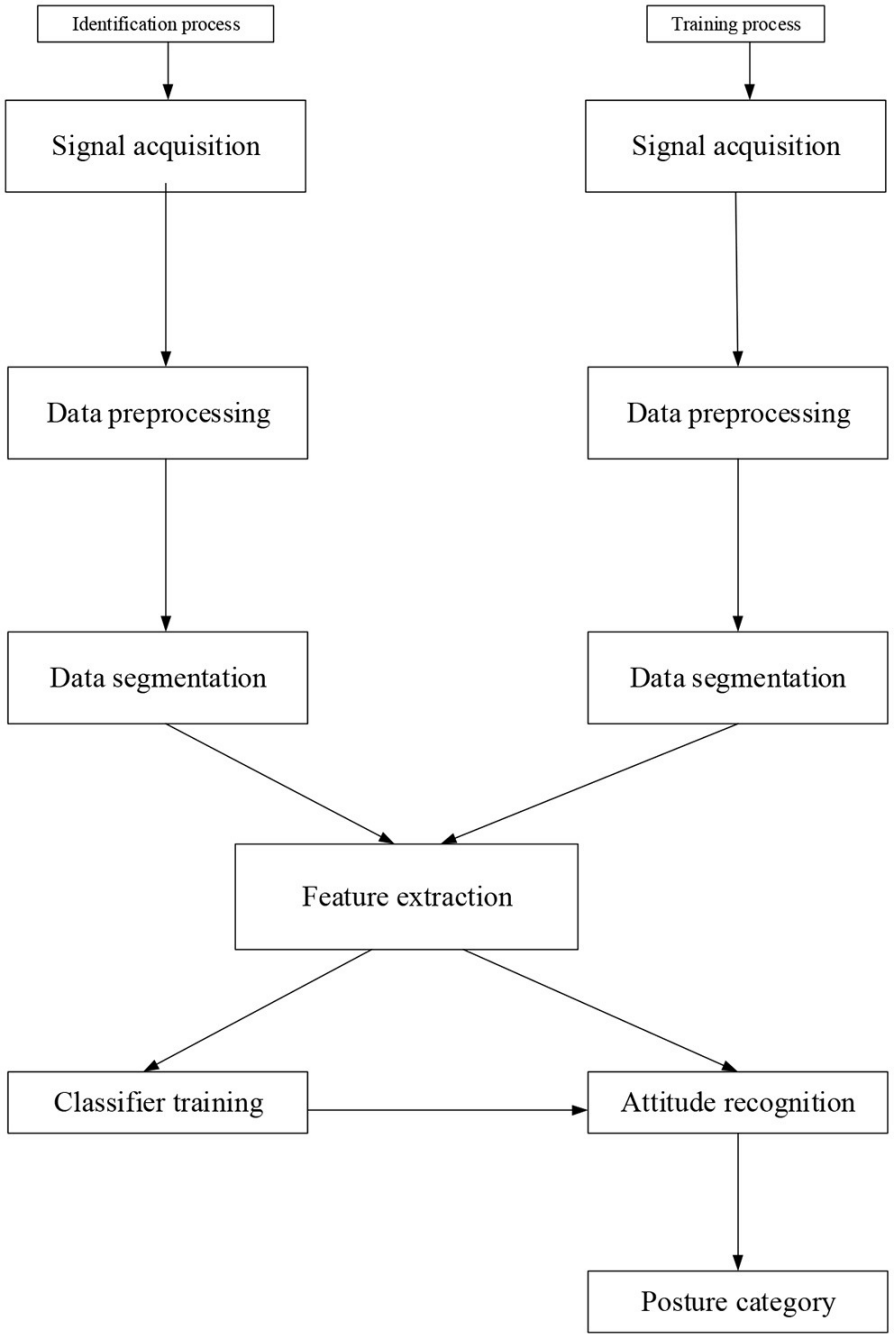
Method flow of human posture recognition.

As a branch of pattern recognition, human attitude recognition has been widely studied and developed in recent years. Human body recognition based on inertial sensors has gradually become a research hotspot. Based on pattern recognition, many scientists have applied imaging technology to the human body recognition of handheld devices (Zhang and Shi, 2021). Figure 1 shows inertial sensors' human body recognition process, comprising specific steps of data acquisition, data processing, data segmentation, feature extraction, and classification training. Especially, in the data acquisition stage, it mainly includes physical or physiological signals, such as acceleration and angular velocity, heart rate, and body temperature. These are primarily covered by detection devices. The data processing can purify and standardize the data to meet the system's requirements. In data processing, data are extracted and analyzed in time and frequency range, separately. In the stage of function extraction, the analysis of element function is basically completed, and the extraction of associated attributes is taken as the sample data of calculation. Selected samples are formed according to the different classification principles, which will lead to the separation of unknown samples (Sannino et al., 2019; Xu and Yi, 2021).

The recognition technology based on image analysis also has many defects, such as the high precision requirement for machines, large size, and unsuitability for wearing. It is difficult to observe in some areas, and the monitoring coverage is limited. Although a large amount of collected data may lead to insufficient storage, real-time monitoring cannot be realized (Soferman, 2019). The development of science and technology based on inertial sensors has promoted the progress of sensor technology, such as smaller size, higher precision, better flexibility, high environmental protection requirements, high sensitivity, low energy consumption, and good real-time performance. It has become the best way to understand the human condition and has been widely used in sports and other fields. Many inertial sensor devices form a spatial network and are widely used (Liu, 2020).

### Data Preprocessing

In the data collection stage, the data signal collected by the sensor device is usually interfered with by the outside world or itself. Interferences include: (1) the jitter generated by the body or periodic signals generated by the surrounding environment during human movements. (2) There are measurement errors in the signal acquisition equipment itself. (3) During the movement, the signal caused by the position deviation of the node is inaccurate. The collected raw data cannot be directly used for analysis and calculation in practical applications. The signal needs to be preprocessed to obtain a more accurate signal after it is ordered. Standard preprocessing methods include denoising and normalization. The two processes of preprocessing will be introduced.

#### Denoising

The original data signals collected by the detection equipment often contain noise signals from an external environment, which is inevitably inaccurate. The signal denoising method in software design is generally called digital filtering, mainly composed of two types of filters: classical filter and current filter (Taborri et al., 2021). When the useful signal and the noise signal are in different frequency bands, adding another noise signal into the linear system can eliminate the noise signal. Commonly used high- and low-pass filters are established according to the principle of the distribution of signals in different frequency bands (Zhao et al., 2020). Still, the traditional filters have some defects and are no longer suitable for signal noise and frequency bands. Modern filters decompose proper signals and sound into random movements. The corresponding autocorrelation function is used to determine the beneficial signal or noise, such as the statistics of the autocorrelation spectrum. Commonly used filtering algorithms are Kalman and Wiener (Estevez et al., 2019).

#### Standardization

Standardization also belongs to normalization, which is the basic step of data mining and plays an essential role in simplifying the calculation. The normalization method is also adopted in human posture recognition. For data processing (Graham et al., 2019), due to the different positions of nodes in the network system of the human body domain, the nodes will shake with the movement of the human body. Data standardization can eliminate the dimensional influence between different data and solve the problem of data comparability. Specifically, their expressions are converted into different data for a comprehensive comparison and evaluation (Hussein et al., 2020). Two data standardization methods are described in detail as follows.

##### Linear Function Transformation

The linear function transformation maps the initial parameters into the interval [0,1] through a linear transformation. Its calculation method are presented as follows:


(1) $$\begin{array}{l} X_{norm}=\frac{X-X_{\text{min}}}{X_{\text{max}}-X_{\text{min}}} \end{array}$$

In Equation 1, *X*_norm_ represents the result of linear function after conversion operation, *X* refers to the initial parameter, *X*_max_ stands for the largest data in the selected sample, and *X*_min_ means the smallest data in the selected sample.

##### Zero Mean Standardization

The zero mean standardization method processes the original data into a normal distribution set with a mean of 0 and a variance of 1. The specific calculation method is defined as:


(2) $$\begin{array}{l} y=\frac{x-\mu }{\delta }, \end{array}$$

where *x* represents the data set, *y* refers to the result after calculation, μ stands for the initial parameter mean, and δ denotes the variance of the initial parameter.

Here, in calculating the node attitude, the three data of angular velocity, acceleration, and magnetic field strength are fused to achieve a more accurate attitude calculation and reduce the noise interference of the sensor. Quaternion means space attitude. Extended Kalman filtering is selected to implement data fusion. The accuracy of the posture solution result is improved. Quaternion and the principle of extended Kalman filtering are introduced in detail.

In 1843, William Rowan of Hamilton created the mathematical concept of Quaternion. It is a simple, super complex number to describe the rotation of a rigid body. Quaternion combines an actual number and three imaginary number units, as shown in Equation 3:


(3) $$\begin{array}{l} Q=w+xi+yj+zk, \end{array}$$

where *w, x, y*, and *z* are real numbers, *i, j*, and *k* are the three imaginary units. A quaternion can also be represented by (*w, x, y, z*). *Q* satisfies Equation 4:


(4) $$\begin{array}{l} w^{2}+x^{2}+y^{2}+z^{2}=1, \end{array}$$

where *Q* is called the unit Quaternion. The unit Quaternion (1, 0, 0, 0) describes the attitude of the rigid body at rest. Only, the unitized Quaternion can describe the rotation of a rigid body, therefore the Quaternion is normalized. Its normalized form is shown in Equation 5:


(5) $$\begin{array}{l} Q_{\text{nwn}}=\frac{Q}{\sqrt{w^{2}+x^{2}+y^{2}+z^{2}}}, \end{array}$$

where *Q*_norm_ means Quaternion after being normalized.

The unit Quaternion can describe the rotation of a rigid body in a three-dimensional (3D) space, as shown in Equation 6. Equation 6 is also Quaternion's differential equation:


(6) $$\begin{array}{l} \dot{Q}=0.5\cdot Q\cdot p, \end{array}$$

where *p* stands for Quaternion. It consists of the angular velocity detected by the gyroscope. The actual part is 0. $\dot{Q}$ is the derivative of Quaternion concerning time, as shown in Equation 7:


(7) $$\begin{array}{l} p=0+\omega_{x}i+\omega_{y}j+\omega_{z}k, \end{array}$$

(8) $$\begin{array}{l} \dot{Q}=a+bi+cj+dk. \end{array}$$

Applying Equations 3, 7 are brought into Equation 6 for calculation according to the complex number arithmetic, as shown in Equation 9:


(9) $$\begin{array}{l} \left[\begin{array}{c} a\\ b\\ c\\ d \end{array}\right]=0.5\left[\begin{array}{c} -x\omega_{x}-y\omega_{y}-z\omega_{z}\\ w\omega_{x}-z\omega_{y}+y\omega_{z}\\ z\omega_{x}+w\omega_{y}-x\omega_{z}\\ -y\omega_{x}+x\omega_{y}+w\omega_{z} \end{array}\right]\\ \;=0.5\left[\begin{array}{cccc} 0 & -\omega_{x} & -\omega_{y} & -\omega_{z}\\ \omega_{x} & 0 & \omega_{z} & -\omega_{y}\\ \omega_{y} & -\omega_{z} & 0 & \omega_{x}\\ \omega_{z} & \omega_{y} & -\omega_{x} & 0 \end{array}\right]\left[\begin{array}{c} w\\ x\\ y\\ z \end{array}\right] \end{array}$$

Equation 9 reflects the relationship between the angular velocity of the rigid body carrier and the derivative of Quaternion concerning time. According to the original state Quaternion, the Quaternion in the new state can be obtained. The normalization process can bring the unit Quaternion used to describe the transformation of a rigid body from one posture to the next. The updated equation is shown in Equation 10:


(10) $$\begin{array}{l} Q_{k+1}=Q_{k}+\;\Delta \;t\cdot {\dot{Q}}_{k}, \end{array}$$

where *k* is a nonnegative integer, representing the time of the system, *Q*_*k*_, and *Q*__*k*_+1_ represent the unit Quaternion of the rigid body posture at the *k*th and *k* + *1*th moments, respectively, represents the derivative of Quaternion for time at the *k*th moment, and Δ* t* represents the time interval between the two samples. As the value of Δ* t* is small, it is assumed that the rigid body rotates at a constant speed in it.

${\dot{Q}}_{k}$ represents the derivative of Quaternion concerning time at the *k*th moment. According to Equation 6, Equation 11 can be obtained as


(11) $$\begin{array}{l} {\dot{Q}}_{k}=0.5\cdot Q_{k}\cdot p_{k} \end{array}$$

Equation (11) is brought into Equation 10, and Equation 12 is obtained:


(12) $$\begin{array}{l} Q_{k+1}=Q_{k}+0.5\cdot Q_{k}\cdot p\cdot \;\Delta \;t \end{array}$$

When the initial state of the system and the rotational angular velocity of the rigid body are known, the Quaternion of the system's state is obtained from Equation 12 to determine the current posture of the rigid body.

Expand Kalman filtering is converted from the nonlinearly changing scene into a linearly changing state for the solution. A non-linearly changing differential equation set is defined to apply the extended Kalman filtering technology to the actual location of nonlinear changes, as shown in Equation 13:


(13) $$\begin{array}{l} x=f\left(x\right)+w, \end{array}$$

where *x* is the state vector of the system. The function *f* (*x*) can calculate the predicted value of the system state at the next moment through the current system state. *w* represents the process noise vector expected to be 0.

The process noise matrix *C* is composed of the process noise vector *w*, as shown in Equation 14:


(14) $$\begin{array}{l} C=E\left(ww^{T}\right) \end{array}$$

The measurement equation is a nonlinear state equation, as shown in Equation 15:


(15) $$\begin{array}{l} z=h\left(x\right)+v \end{array}$$

The function *f* (*x*) is the observation equation of the system. *v* is the measurement error vector expected to be 0, represented by the measurement noise matrix *R*, as shown in Equation 16:


(16) $$\begin{array}{l} R=E\left(vv^{T}\right) \end{array}$$

As the measurement process is a nonlinear changing equation, *k* is used to represent the time. Equations 13, 15 are transformed into Equations 17, 18, respectively:


(17) $$\begin{array}{l} z_{k}=h\left(x_{k}\right)+V_{k} \end{array}$$

(18) $$\begin{array}{l} x_{k+1}=f\left(x_{k}\right)+W_{k} \end{array}$$

Assuming that the current optimal estimation state of the system is, the prior error $e_{k}^{-}$ of the system can be obtained according to the expected state value of the system, as shown in Equation 19:


(19) $$\begin{array}{l} e_{k}^{-}=x_{k}-{\hat{x}}_{k}^{-} \end{array}$$

The posterior error of the system is shown in Equation 20:


(20) $$\begin{array}{l} e_{k}=x_{k}-{\hat{x}}_{k} \end{array}$$

Knowing that the prior error of the system is the expectation of the covariance of the system error, the covariance of the preceding error is shown in Equation 21:


(21) $$\begin{array}{l} P_{k}^{-}=E\left(e_{k}^{-}e_{k}^{-T}\right) \end{array}$$

The covariance of the posterior error is shown in Equation 22:


(22) $$\begin{array}{l} P_{k}=E\left(e_{k}e_{k}^{T}\right) \end{array}$$

According to the principle of the extended Kalman filtering algorithm, the expected value of the next moment of the system can be obtained by the optimal estimation of the current system. The desired correction of the system state is completed by mapping the system's measurement error to the system's state domain, thereby obtaining the optimal estimation of the system at the next moment. The state of the system is estimated to be continuously updated. The best estimate of each moment of the system is obtained. The Kalman filtering method of the nonlinear system is shown in Equations 23–27:


(23) $$\begin{array}{l} {\hat{x}}_{k+1}^{-}=f\left({\hat{x}}_{k},u_{k}\right), \end{array}$$

(24) $$\begin{array}{l} P_{k+1}^{-}=F_{k}P_{k}^{-}F_{k}^{T}+C, \end{array}$$

(25) $$\begin{array}{l} K_{k+1}=P_{k+1}^{-}H_{k+1}^{T}{\left(H_{k+1}P_{k+1}^{-}H_{k+1}^{T}+R\right)}^{-1}, \end{array}$$

(26) $$\begin{array}{l} {\hat{x}}_{k+1}={\hat{x}}_{k+1}^{-}+K_{k+1}\left(z_{k+1}-h\left({\hat{x}}_{k+1}^{-}\right)\right), \end{array}$$

(27) $$\begin{array}{l} P_{k+1}=P_{k+1}^{-}-K_{k+1}H_{k+1}P_{k+1}^{-} \end{array}$$

Equation 23 represents the desired state at the next moment. It can be obtained from the well-estimated state at the current moment. In Equation 24, *F* is the Jacobi matrix of the function *f* (^.^). This equation expresses that the current error covariance estimate is obtained through the posterior error covariance matrix now. Equation 25 gives the method of solving Kalman gain *K*_*k*_. *H*_*k*_ is the Jacobi matrix of the function. Equation 26 combines the expected value of the state at the next moment and the observed value. It obtains the Kalman gain, thereby obtaining the optimal estimation value of the form at the current moment. Kalman filtering is an iterative cycle process. Therefore, the updated test error covariance is used for future iterations. Equation 27 uses the prior error covariance at the next moment to update. It is brought into Equation 24 for a new round of iterations.

The expansion of Kalman filtering mainly includes two processes: time iteration and measurement iteration. Time iteration includes Equations 23, 24, which completes the prediction of system state and error covariance primarily. Measurement iteration uses observations to correct expectations, to obtain more accurate estimates, and to obtain optimal forecast, as shown in Equations 25–27. In Equation 26, Kalman gain is mainly used to weigh the state expectation and the proportion of the system measurement value in calculating the optimal estimation. The greater the Kalman gain, the closer the optimal estimate is to the observed value.

In actual situations, the process noise *w*_*k*_ and the measurement noise *v*_*k*_ conform to the white noise of the normal distribution. The two types of noise are not correlated with each other. Therefore, the covariance matrix *C* of the process noise and the covariance matrix *R* of the measurement noise are constants. At the initial moment of the system, the posterior error covariance matrix *P*_0_ of the system can be set to any diagonal matrix as the initial value. The initial values of other states of the system can be set arbitrarily. In the iterative process of the system, these values will automatically converge and get closer to the actual situation.

#### Quaternions Method

In 1843, the British mathematician W.R. Hamilton introduced Quaternions in mathematics. However, until the late 1960's, this method had not been put into practical use. With the development of space technology and strapdown inertial navigation system (SINS) technology, Quaternions have attracted people's attention. Solving the Quaternions differential equations involves solving four differential equations. Although this is one more equation than solving Euler's differential equations, it has the advantages of less computation, high precision, and the avoidance of singularities. This method is one of the focuses of current research. Due to the direction cosine method, skew, scale, and drift errors will be generated when solving the attitude dynamics of the carrier. However, it is essential in SINS to estimate these errors when doing attitude solutions. The advantage of the Quaternions method compared to the directional cosine method is that the skew error is equal to 0. The derivation of the scale error leads to an analytical expression that facilitates further analysis. The directional cosine method can only analyze and detect scale errors in exceptional cases without drawing general conclusions. A comparison of the Euler angle method, directional cosine method, and Quaternions method from different angles shows that Quaternions method performs the best.

#### Kalman Filtering

Kalman filtering does not require the assumption that both signal and noise are stationary processes. For the system disturbance and observation error (i.e., noise) at each moment, if some appropriate deductions are made about their statistical properties, and by processing the observation signal containing noise, the minimum error can be obtained in the average sense. Therefore, the Kalman filtering theory is applied in communication systems, power systems, aerospace, environmental pollution control, industrial control, radar signal processing, and other industries and has achieved many successful application results. For example, in image processing, Kalman filtering restores the images blurred by some noise. After making some statistical assumptions about the noise, the Kalman filtering algorithm can be used to obtain the actual image with the minor mean square error from the blurred image in a recursive way, so that the blurred vision can be restored.

### Classification Algorithm

#### Decision Tree

Decision tree (DT) is a monitoring learning method, which is often used for data classification and regression. It is the essence of DT to simplify complex problems into a hierarchical structure to solve these problems, therefore it is also a multilevel decision model. DT can be regarded as a tree structure composed of nodes and sharp edges, a theoretical statistical model (Rampersad, 2020). The node types include leaf and internal nodes. The internal nodes represent the recognition of specific attributes of multiple samples, the extended branches stand for the recognition results, and the leaf nodes denote the specific classification results. The construction of DT is complex. Feature selection and partition is the primary step of constructing DT, in which feature selection contains detailed information, which is mainly based on some indicators related to characteristics. The most common hands are gain rate and information gain and inversion (Senhaji et al., 2021). Feature segmentation can also be regarded as a re-segmentation method. Below them, different categories can be filtered so that every data pointing to an edge has the same type as possible. Standard DT construction algorithms are C4.5, Iterative Dichotomiser 3 (ID3), Classification and Regression Trees (CART), etc. The principle of DT is relatively simple, the construction process is not complicated, and the construction time is relatively short (Kotter and Ranschaert, 2021). However, this method is not suitable for the problem of missing data, and it is prone to overfitting.

#### Naive Bayes

Naive Bayes (NB) method is a simple classification method. Based on the Bayesian theory, the classification probability of each type of sample is calculated, and the most probable category is selected as the classification result. Simple Bayesian algorithm has a strong theoretical foundation and is a relatively stable classification method based on classical mathematical theory. Because this method is simple and insensitive to lost data, there are some limitations in practical applications, which need to be improved to make the models independent of each other. These problems are usually encountered (Радутний, 2019). In the application of human identification markers, the occurrence of events is uncertain, therefore it is difficult to obtain the prior probabilities of different classification behaviors, hence the Bayesian algorithm is not applicable.

#### Support Vector Machine

Support vector machine (SVM) refers to a supervised classification method, which is widely used in machine learning at present. It was first used in 1960 and applied to the optimal classification hyperplane. The basic principle of SVM is its minimal structure, therefore it has strong generalization ability. Initially, this method was mainly used to deal with the classification of two simple categories. It can automatically find out the vector machine to achieve the optimal classification, which is linear. SVM uses the kernel technique to make it applicable to nonlinear classification. Multiple SVMs are combined to meet the requirements (Ha et al., 2019). SVMs are constantly being optimized and improved. The SVM algorithm is widely used in text classification, image classification, handwriting font recognition, and other fields.

#### Artificial Neural Network

It is a machine learning algorithm that imitates the biological neural network model, which has a complex network structure. Each neuron has a simple structure and unique function. This is an adaptive nonlinear information processing system. Artificial neural network (ANN) can be summarized as a three-layer mathematical model, including an input layer, an output layer, and a hidden layer (Luo et al., 2019). The input layer consists of many neurons for receiving information. The hidden layer is also called as concealed layer. The neural network can be divided into input and output layers. It is widely used in image processing and language recognition. A neural network has strong self-learning and nonlinear adaptive ability and has relatively little interference to noise data.

## Research Model and the Methodology

### Recognition of Basketball Players' Actions During Training

#### The Definition of Basketball Posture

**Figure 2:**
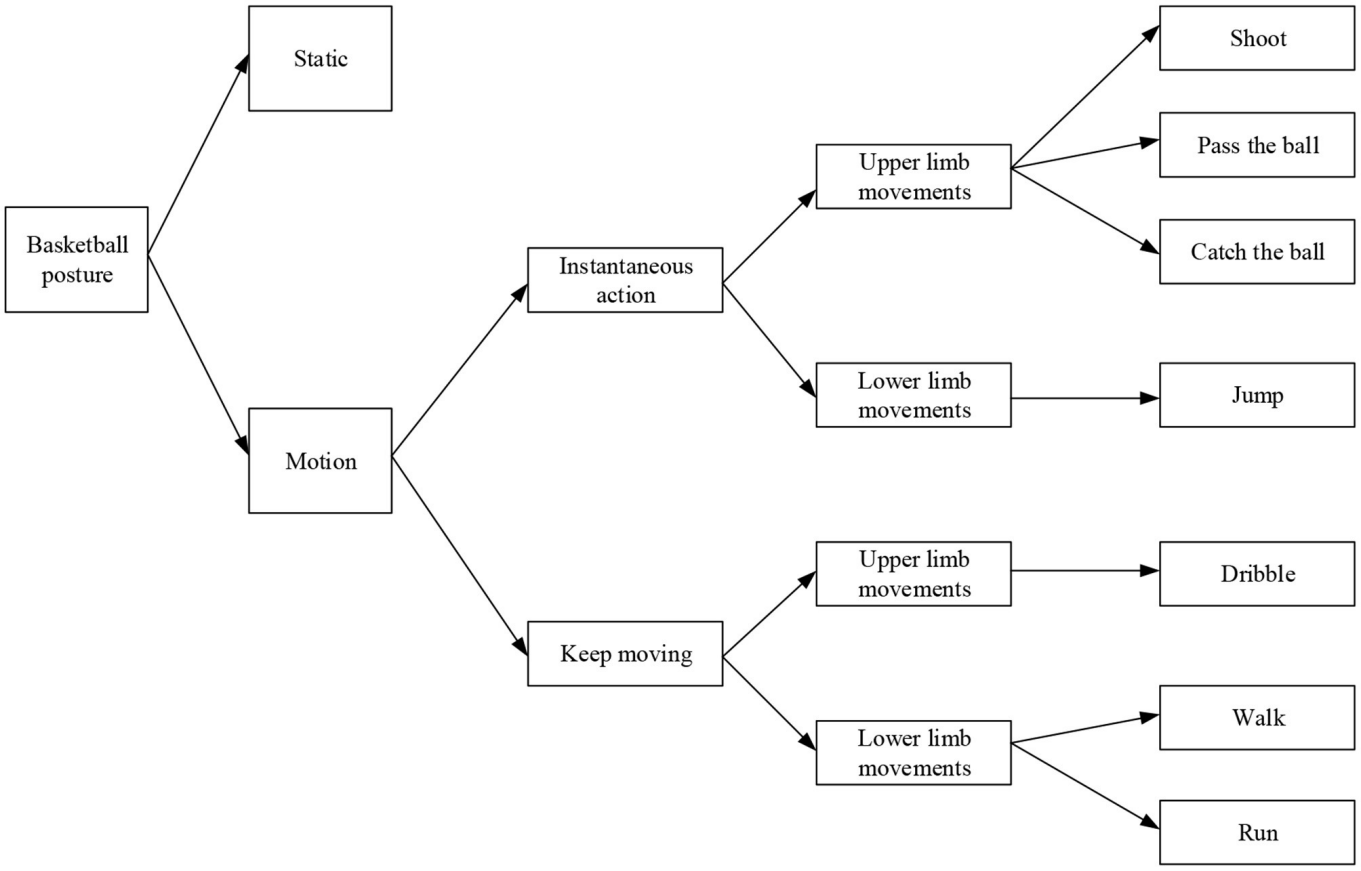
Composition of basketball training action.

When basketball players train, they usually perform complex skill actions. Figure 2 presents an analysis of the composition of skill actions in the training process of basketball players. According to the different states of different limbs at a certain moment, the athletes' action states in basketball training can be simply divided into two types: motion and static. Under the static state, the movement of limbs of athletes belongs to a stationary state, and the relative movement state refers to the state when the limbs perform related exercises. For example, when the athletes perform the ball-catching action, the legs of professional athletes will not change, therefore the legs at this time belong to a static state, while the upper limbs participate in the ball-catching action. Therefore, the arm is in motion (He, 2021). The shooting, catching, passing, and dribbling of upper limbs and jumping, walking, and running of lower limbs are defined as unit movements. In the motion state, the exercises can be divided into rapid movements and continuous movements according to whether they are periodic or not. Generally, instantaneous action does not have periodicity. It only contains one-unit action, such as catching, dribbling, and shooting. In this way, the continuous basketball training action is periodic. There will be many small unit actions when completing a set of training actions, such as constant walking, dribbling, and running dribbling. Therefore, in recognizing athletes' movements in basketball training, it is imperative to distinguish the activities of upper and lower limbs. Therefore, a division method based on unit action extraction is adopted here.

#### Data Segmentation

**Figure 3:**
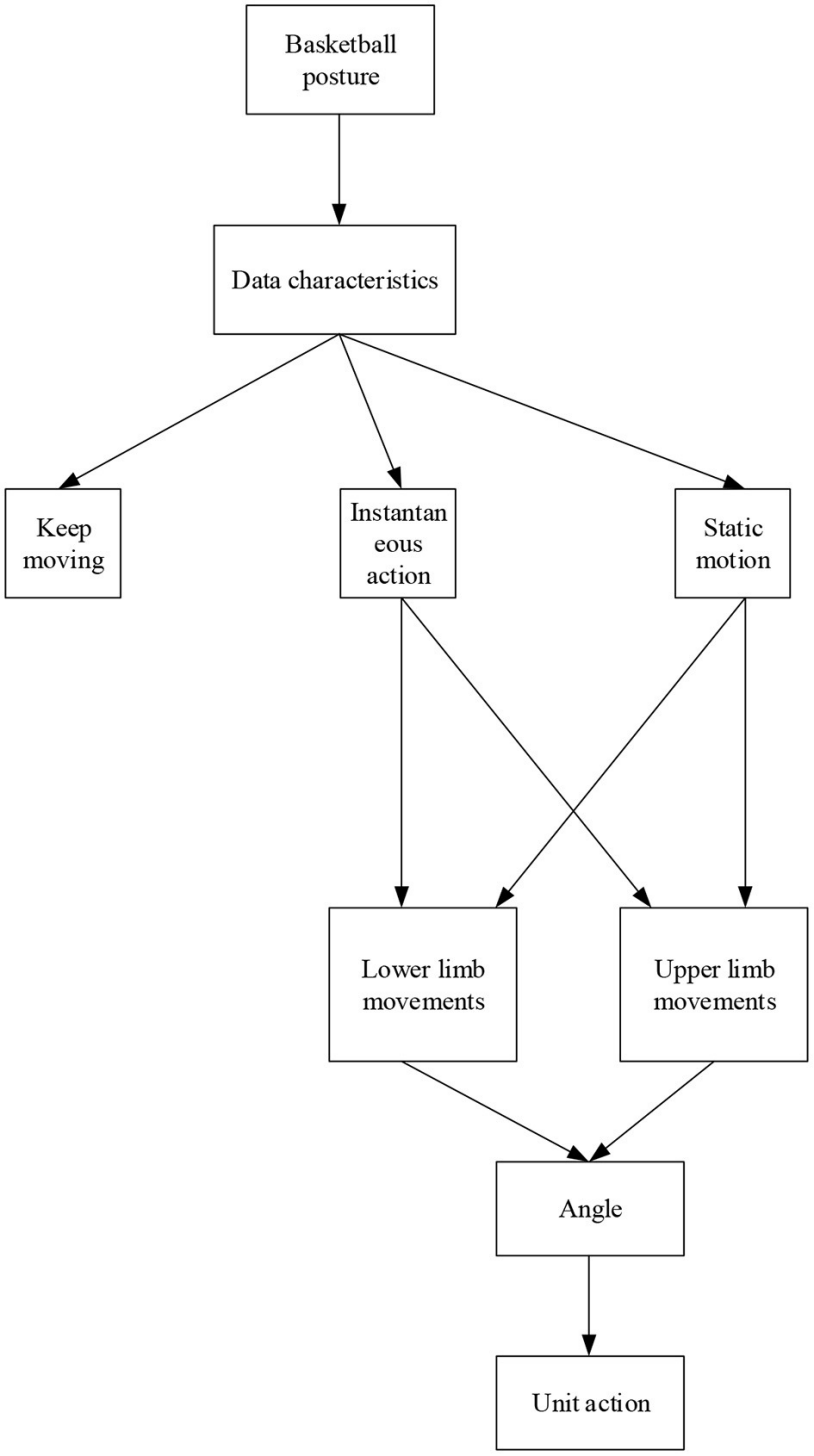
Data division method of basketball posture.

Data segmentation is generally divided into two stages, and Figure 3 shows the specific data partitioning process. In the data division of the first stage, according to the discreteness characteristics of action data in basketball training of athletes in two different states, what is extracted are the athletes' action data and the sustained and instantaneous actions of athletes in a series of activities. Because there are many athletes' persistent movements and much need of unit movements, in the second stage of data division, according to the changes in the limbs angles of basketball players during the training process, it is finally realized of the data extraction of athletes' continuous movements (Dudnik et al., 2021).

#### Division of Exercise Status

The degree of dispersion indicates the degree of difference between the values of the observed variable. The difference between the sensor signal sample values is taken as the dispersion. Taking angular velocity as an example, $\omega_{n}^{x}$ represents the *x*-axis angular velocity data at the *n*th time. $\omega_{n-1}^{x}$ represents the *x*-axis angular velocity data at the *n* – 1th time. $d_{n}^{x}$ represents the difference between the angular velocity of the *x*-axis of the sensor at the *n*th moment and the angular velocity at the last moment. The dispersion $d_{n}^{x}$ can be obtained by Equation 28:


(28) $$\begin{array}{l} d_{n}^{x}=\left|\omega_{n}^{x}-\omega_{n-1}^{x}\right| \end{array}$$

The motion data includes angular velocity data and acceleration data. The data characteristics of each sensor need to be considered comprehensively to achieve the accurate division of actions. $D_{n}^{x}$ represents the dispersion of the acceleration sensor data at the *n*th time. $d_{n}^{g}$ represents the dispersion of the angular velocity sensor data at the *n*th time. represent the dispersion of the acceleration and angular velocity of each axis, and the expressions of $D_{n}^{a}$ and $D_{n}^{g}$ are shown in Equations 29, 30:


(29) $$\begin{array}{l} D_{n}^{a}=d_{n}^{a_{s}}+d_{n}^{a_{y}}+d_{n}^{a_{z}}, \end{array}$$

(30) $$\begin{array}{l} D_{n}^{g}=d_{n}^{g_{s}}+d_{n}^{g_{y}}+d_{n}^{g_{z}} \end{array}$$

In the stationary state, the acceleration and angular velocity dispersion are kept below the threshold values λ_*a*_ and λ_*g*_, respectively. In the state of exercise, the sensor data will change rapidly with the athlete's movements. Dispersion can reflect the degree of difference in the sensor data. Therefore, the dispersion feature can realize the athlete's limb state division. γ_*n*_ represents the state of the athlete's limb at the *n*th moment. When γ_*n*_ is 0, it indicates a static state. When γ_*n*_ is 1, it represents the state of motion, as shown in Equation 31:


(31) $$\begin{array}{l} \gamma_{n}=\{\begin{array}{c} 0,{\text{D}}_{n}^{a}<\lambda_{a}\;{\text{and D}}_{n}^{g}<\lambda_{g}\\ 1,{\text{D}}_{n}^{a}\geq \lambda_{a}\;{\text{or D}}_{n}^{g}\geq \lambda_{g} \end{array} \end{array}$$

The data dispersion degree of each sensor is calculated, and the threshold value can identify each movement state.

#### Division of Unit Actions

By dividing the action state, instantaneous action and continuous action are obtained. The division of unit actions is the further processing of constant measures. In constant movement, the movements of the legs and arms are in constant periodic changes, and the periodicity of the continuous changes is more pronounced. Therefore, it is feasible to realize the division of unit actions based on the movement data of the arms and legs. Through data comparison, the angular velocity data can intuitively describe the angular change during the movement of the rigid body. Angular velocity is used as a reference, and the data are divided as a reference.

The degree of dispersion represents the degree of difference between the values of the observed variable. The difference between the sample values of the sensor signal is used as the dispersion. Taking the angular velocity as an example, $\omega_{n}^{x}$ represents the *x*-axis angular velocity data at the *n*th time, $\omega_{n-1}^{x}$ represents the *x*-axis angular velocity data at the *n* – 1th time, and $d_{n}^{x}$ represents the difference between the angular velocity of the sensor *x*-axis at the *n*th moment and the angular velocity of the last moment. The degree of dispersion $d_{n}^{x}$ is shown in Equation 32:


(32) $$\begin{array}{l} d_{n}^{x}=\left|\omega_{x}^{n}-\omega_{n-1}^{x}\right| \end{array}$$

The motion data includes angular velocity data and acceleration data. Each sensor data feature is comprehensively considered to achieve an accurate division of actions. $d_{n}^{a}$ represents the dispersion of the acceleration sensor data at the *n*th time, and $d_{n}^{g}$ represents the dispersion of the angular velocity sensor data at the *n*th time. $D_{n}^{a}$ and $D_{n}^{g}$ are shown in Equations 33, 34:


(33) $$\begin{array}{l} D_{n}^{a}=d_{n}^{a_{x}}+d_{n}^{a_{y}}+d_{n}^{a_{z}} \end{array}$$

(34) $$\begin{array}{l} D_{n}^{g}=d_{n}^{g_{x}}+d_{n}^{g_{y}}+d_{n}^{g_{z}} \end{array}$$

At rest, the acceleration and angular velocity dispersions remain below the thresholds λ_*a*_ and λ_*g*_, respectively. In the state of motion, the sensor's data will change rapidly with the athlete's movement, and the degree of dispersion can reflect the degree of difference of the sensor data. Therefore, the discrete feature can realize the athlete's limb state division. γ_*n*_ represents the state of the athlete's limb at the *n*th moment, as shown in Equation 35:


(35) $$\begin{array}{l} \gamma_{n}=\{\begin{array}{c} 0,D_{n}^{a}<\lambda_{a}\;and\;D_{n}^{g}<\lambda_{g}\\ 1,D_{n}^{a}\geq \lambda_{a}\;and\;D_{n}^{g}\geq \lambda_{g} \end{array} \end{array}$$

**Figure 4:**
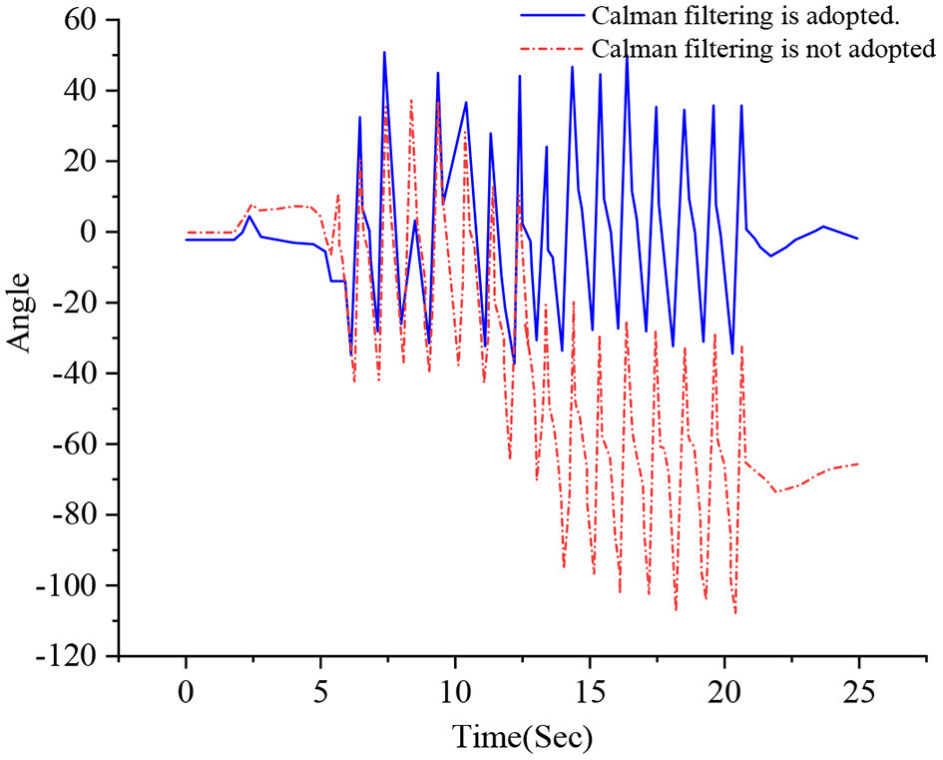
A comparison of Kalman filtering effects of changing the walking angle.

In basketball, the sensor signal is easily affected by the human body and the external environment. When calculating the angle during limb movement, the Kalman filtering algorithm is used to fuse acceleration, magnetic field strength, and angular velocity data to reduce the influence of external noise. Figure 4 shows the change curve of the calf angle during walking. The abscissa represents time, and the ordinate represents the calf angle. The dotted line is the angle curve obtained without the Kalman filtering algorithm, periodically changing. After a period, the angle value shifted significantly. The solid line is the angle curve obtained by the Kalman filtering algorithm, which fluctuates at the same amplitude on both sides of 0°. Kalman filtering algorithm processing data can reduce the interference of noise signals.

Figure 4 uses the Kalman filtering algorithm to process data whose value remains at [−40, 60]. The data values that are not processed by the Kalman filtering algorithm remain at [−110, 40], which is less stable than the former data because the Kalman filtering algorithm can effectively remove the interference data and ensure the accuracy of the obtained data.

#### Posture Feature Extraction of Basketball Players

Data division obtains the unit action data composed of acceleration and angular velocity. $a_{n}^{x}$, $a_{n}^{y}$, and $a_{n}^{z}$ represent the accelerations of the three axes of the *n*th sampling point, and, $g_{n}^{y}$, and indicates the angular velocity of three axes at the *n*th sampling point. Parameters *a*_*n*_ and *g*_*n*_ express the acceleration vector sum and angular velocity vector sum, respectively, as shown in Equations 36, 37:


(36) $$\begin{array}{l} a_{n}=\sqrt{{\left(a_{n}^{x}\right)}^{2}+{\left(a_{n}^{y}\right)}^{2}+{\left(a_{n}^{z}\right)}^{2}}, \end{array}$$

(37) $$\begin{array}{l} g_{n}=\sqrt{{\left(g_{n}^{x}\right)}^{2}+{\left(g_{n}^{y}\right)}^{2}+{\left(g_{n}^{z}\right)}^{2}} \end{array}$$

The eight-dimensional vector is mainly composed of combined acceleration, triaxial acceleration, combined angular velocity, and triaxial angular velocity. And, *n* is the number of points that unit basketball training movements can collect, each dimension in the vector contains the sampling data of *N* basketball training actions. And, each basketball training action is taken as a sample. Every sample will become an N^*^8-dimensional matrix. Simultaneously, every basketball training action should be calculated in all dimensions, therefore it is necessary to calculate the signal, which mainly has two signal features: frequency and time. There are two main features in the time domain: variance and mean. Parameters μ_*a*_ and δ^2^ represent the mean and variance of acceleration components in basketball training actions, which are obtained by Equations 38, 39 (Shivale et al., 2020):


(38) $$\begin{array}{l} \mu_{a}=E\left(a\right)=\frac{1}{N}\sum_{i=1}^{N}a_{i} \end{array}$$

(39) $$\begin{array}{l} \delta^{2}=\frac{1}{N}\sum_{i=1}^{N}{\left(a_{i}-\mu_{a}\right)}^{2} \end{array}$$

The frequency-domain features mainly include two types: frequency and signal peak value after the discrete Fourier transform. According to the discrete Fourier transform, the obtained action signal is transformed from the time domain to the frequency domain. *S*_DFT_(*n*) represents the Fourier transform result of the *n*th sampling point, and *j* represents the imaginary unit, and its calculation process is as shown in Equation 40:


(40) $$\begin{array}{l} S_{DFT}\left(n\right)={\sum_{i=0}^{N-1}a_{i}e}^{-j\frac{2\pi }{N}in} \end{array}$$

The peak value *S*_DFT_(*k*) of the signal is obtained by Fourier transform, where *K* represents the sampling point corresponding to each peak during Fourier transform. The frequency *F* in the Fourier transform is calculated by Equation 41, and the sampling frequency is represented by *f*_*x*_:


(41) $$\begin{array}{l} f=K\times \frac{f_{x}}{N} \end{array}$$

Through feature calculation, it can figure out the features of collected action signals in the frequency domain and time domain.

#### Basketball Posture Recognition

The essence of basketball gesture recognition is constructing a classification model that satisfies the classification of basketball action data. For each specific basketball action, after data collection, data preprocessing, data division, and feature extraction, the attribute set of the specified basketball action can be obtained, that is, the feature vector set. These feature vector sets are abstract data sets of basketball actions. The corresponding classification can be obtained in the calculation of the classifier model. The attributes contained in the feature vector are complex. The feature vector feature is selected to eliminate irrelevant and redundant attribute values in the feature vector. The priority search algorithm and principal component analysis method are adopted in attribute screening. Feature selection realizes the dimensionality reduction of feature vectors. This reduces the complexity of the classification calculation process and improves the working efficiency of the system. In the experiment, the sensor nodes are fixed on the calf and forearm of the subject to detect the action and behavior information of different limbs. According to the different placement positions of the nodes, the data set of each kind of movement is divided into the upper and the lower limb movement data set. Different sample sets are separately constructed classifiers to realize the specific division of upper and lower limb unit actions. The upper and lower limb movements are combined, and the basketball movement posture performed by the current examinee is obtained. In constructing the classification model, four commonly used classification algorithms are used: C4.5 DT, SVM, Bayesian network, and backpropagation ANN. The output results of these four classification methods are compared and analyzed. The best classification method following the experimental environment is obtained.

### Intelligent System Design

#### Target Machine Software

It realizes signal transmission by the position calculation module and runs on the nodes of the sensor. The function of the base station is to receive the nodes and collect the parameters of the obtained nodes. Therefore, a serial port is used to transmit the data to the upper computer, and the upper computer uses the serial port to collect the action data of basketball players and perform classified calculations and analysis of actions. The data are passed to the upper computer to shape the athlete's body posture (Echevarría et al., 2019). Data initialization mainly completes Quaternion initialization, acceleration initialization, and magnetic field intensity initialization. The received data mainly includes the position information of each sensor, including acceleration, angular velocity, and magnetic field strength. According to the height calculation method, one-quarter of the data is recursive. In the data fusion process of Kalman filter design, the acceleration, angular velocity, and magnetic field strength determine whether the acceleration data can be used for position calculation when the object position changes (How and Wei, 2019). If the position of the object changes and the acceleration data cannot be used for filtering calculation, the position data are directly sent to a computer through a serial port and converted into a cosine matrix. And then, it is converted into unified coordinates for the upper computer to display.

#### Upper Computer Software

The function of the upper computer is to realize the consignment of athletes' skill movement data and the display of athletes' postures. The upper computer reads the relevant attitude information data sent from the attitude node to the base station through a serial port. And, the upper computer part constructs a 3D model of the human body through visualization toolkit (VTK) development tools, and through processing and calculating the collected posture information, thereby intuitively displaying the posture of the human body.

The upper computer module only receives data of the quaternion data type. According to the OpenGL theory, OpenGL cannot directly utilize and draw quaternions. Therefore, before applying the OpenGL theory, it is necessary to process the obtained data. Therefore, a data processing module is added between the received data and the human body display module (Joshua and Niloufer, 2020). By changing Quaternion into directional cosine and then converting directional cosine into four-dimensional homogeneous coordinates, the combination with OpenGL can be realized after such a transformation.

**Figure 5:**
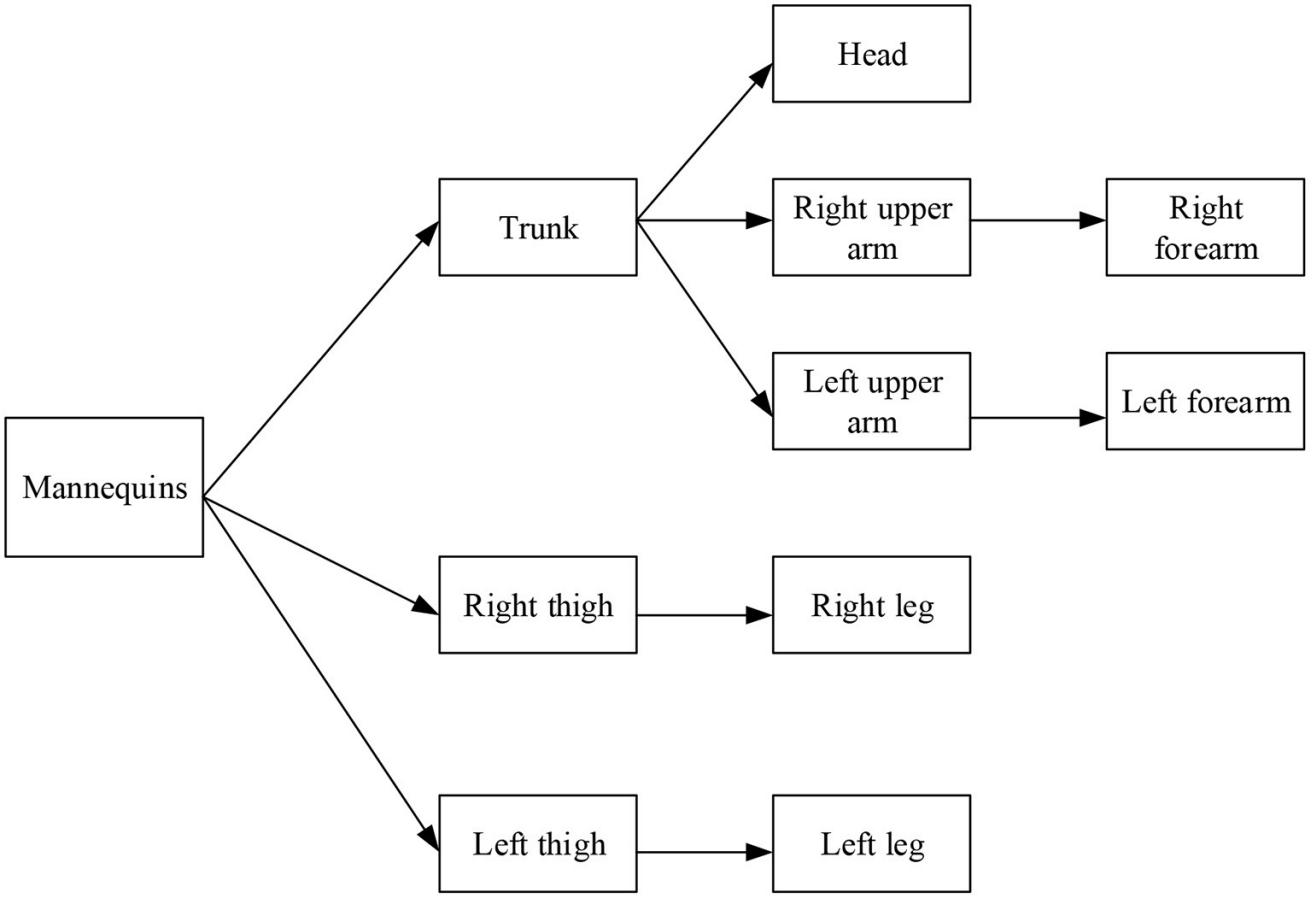
The model of the human body.

The 3D model of basketball elements can be divided into 10 structural parts. In these 10 parts, the bones will produce associated animation effects after rotation and translation due to the interaction of various structures. Figure 5 presents the specific structural relationship. Each node in Figure 5 can be regarded as a rigid body. In the spatial structure, rigid bodies are usually represented by the two points. The rotation centers of these two nodes are the origin, and the other node is called the endpoint. The relationship between two nodes is the parent–child relationship by default. When the parent node rotates, it will drive the child node to translate.

**Figure 6:**
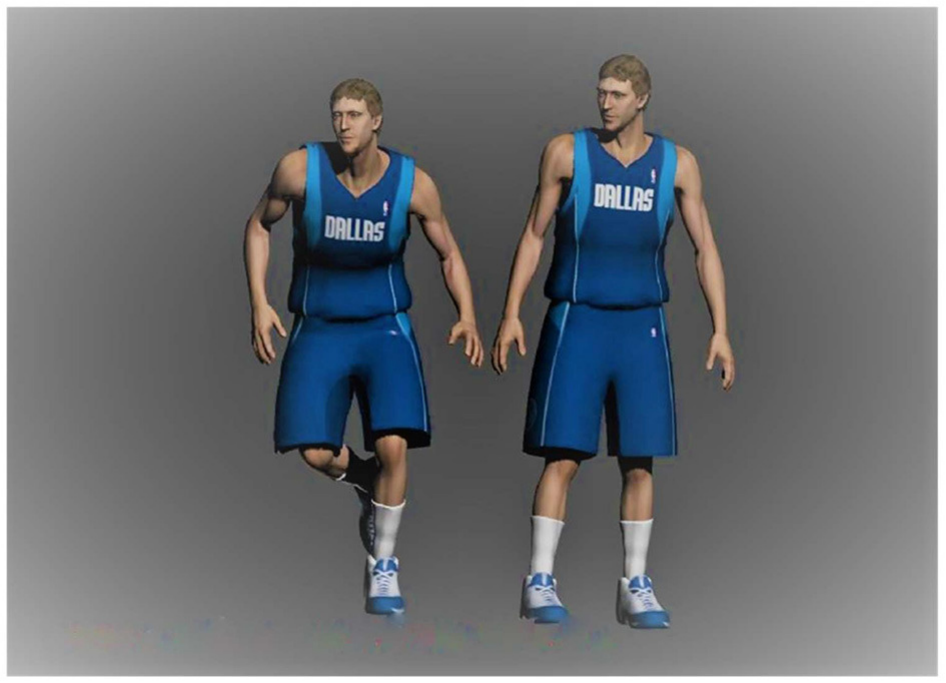
The model of 3dMax basketball player.

In Figure 5, the method of skeletal animation is referred to make that the established basketball player model have animation effects. Geometric vertices are bound to bones, and spirits are generated by controlling the translation and rotation of these bones. The specific performance is that each bone has a weight factor for all vertices, and the size is 0 to 1, where 0 means that the bone is not related to the vertex. Multiple bones may drive a vertex. However, the weights of the bones connecting the vertex should add up to 1. The obj file does not contain bone information, and it is necessary to use 3dMax to export the set weight data in text form. Afterward, 3dMax tools are used to draw the concrete human model and bind the bones. The experiment rotates continuously from 0° to 1,080°, with data sampling every 90°. The investigation is divided into two groups. To compare the compensation effect of Kalman filtering on the angle calculation, the first group of the investigation does not use any compensation method. It directly obtains the rotation angle of the sensor through the angular velocity integration. The second group of experiments uses the extended Kalman filtering method to compensate for the angular output of the sensor nodes. Figure 6 shows the model constructed in 3dMax.

The related methods of skeletal animation to connect the geometric vertices of the model characters are combined with the bones' positions to generate animation by using the rotation and translation of the bones of the characters in the model. Specifically, each bone has a weight for all vertices, ranging from 0 to 1, where 0 indicates that the bone is irrelevant to the vertex. Multiple bones may drive a vertex, but the weights of the bones connecting the vertex should add up to 1.

#### Design of Basketball Assistant Robot

The overall shape of the robot is designed as a humanoid omnidirectional wheeled mobile robot with a relatively simple structure. According to the characteristics and functional requirements of the training land, the overall design plan is: the mechanical system includes three parts: the swinging robotic arm, the robot torso, and the mobile base. A motor drives the mechanical arm to swing up and down at a certain angle, which can expand the defensive area and simply block the pass and pitch. The robot's torso is generally a “bell-shaped” cavity structure with a narrow upper and a lower width to facilitate the installation of the middle and outer protective layers. A gyroscope is installed in the middle and lower part of the torso to keep the robot balanced when it is bumped. The mobile base consists of a driving gear train, a base frame, and base armor. The driving wheel train adopts the vertical layout of four omnidirectional wheels. Four servo motors independently drive the four omnidirectional wheels to meet the requirements of all-round steering of the robot. As the robot needs to sense and defend the position of the ball and players, vision sensors are required. A gyroscope device is installed in the robot body to prevent the robot from being knocked down during training.

During the data collection process of basketball training movements, the basketball teaching assistant robot tested the walking, jumping, running, and dribbling in place without the ball, running dribbling, walking dribbling, passing with the ball, and eight male testers, respectively. Nine kinds of basketball training actions, shooting, and catching are collected for related data. A total of 5,000 samples are collected this time. When dribbling the ball, the upper body movements of athletes mainly include dribbling in place, running dribbling, walking dribbling, passing, shooting, and catching, totaling 2,400. There are 2,600 data records of lower body movements during athletes' training, including walking, jumping, running, dribbling in place, running dribbling, and shooting while dribbling the ball. During the data collection process, each athlete participating in the test must complete all technical movements, and the basketball assistant robot will record the number of basketball technical movements.

## Experimental Design and Performance Evaluation

### Evaluation of the Athlete's Attitude Estimation of the Intelligent System

The evaluation methods of joint human points include percentage of correct keypoints (PCK) and object keypoint similarity (OKS). Here, Euclidean distance is used to compute the similarity between the two poses and the score between 0 and 1 (Wetzel et al., 2020). Euclidean distance is one of the most common distance measures. It measures the absolute distance between all points in a multidimensional space. The Euclidean distance of two *n*-dimensional vectors *X* = (*x*_1_, *x*_2_, *x*_3_, ..., *x*_*n*_) and *Y* = (*y*_1_, *y*_2_, *y*_3_, ..., *y*_*n*_) is shown in Equation 42:


(42) $$\begin{array}{l} D\left(X,Y\right)=\sqrt{\overset{n}{\underset{i=1}{\sum }}{\left(x_{i}-y_{i}\right)}^{2}} \end{array}$$

When the OKS value is more significant than 0.5, the athlete's posture can be correctly detected. Athlete detection and pose estimation are both detection tasks. The evaluation method of the inspection task is selected as the commonly used evaluation index, namely, the maximum recall rate. For experimental results, there are four possible situations between the predicted and the actual values.

TP, FP, FN, TN, and TP indicate that positive samples are predicted to be true. FP means that negative samples are expected to be true. FN means that positive samples are predicted to be false. TN indicates that the pessimistic sample prediction is incorrect. *P* stands for precision, *R* stands for recall, and *S* represents specificity, as shown in Equations 43–45:


(43) $$\begin{array}{l} P=\frac{TP}{TP+FP}, \end{array}$$

(44) $$\begin{array}{l} R=\frac{TP}{TP+FN}, \end{array}$$

(45) $$\begin{array}{l} S=\frac{TN}{FP\;+\;TN} \end{array}$$

Different detection confidence thresholds are set. Different test results will be produced. The detection result of each threshold is calculated as *P* and *R*. The recall obtained is the maximum recall when the confidence threshold is set to the minimum value.

### Data Partition Principle Based on Discreteness

**Figure 7:**
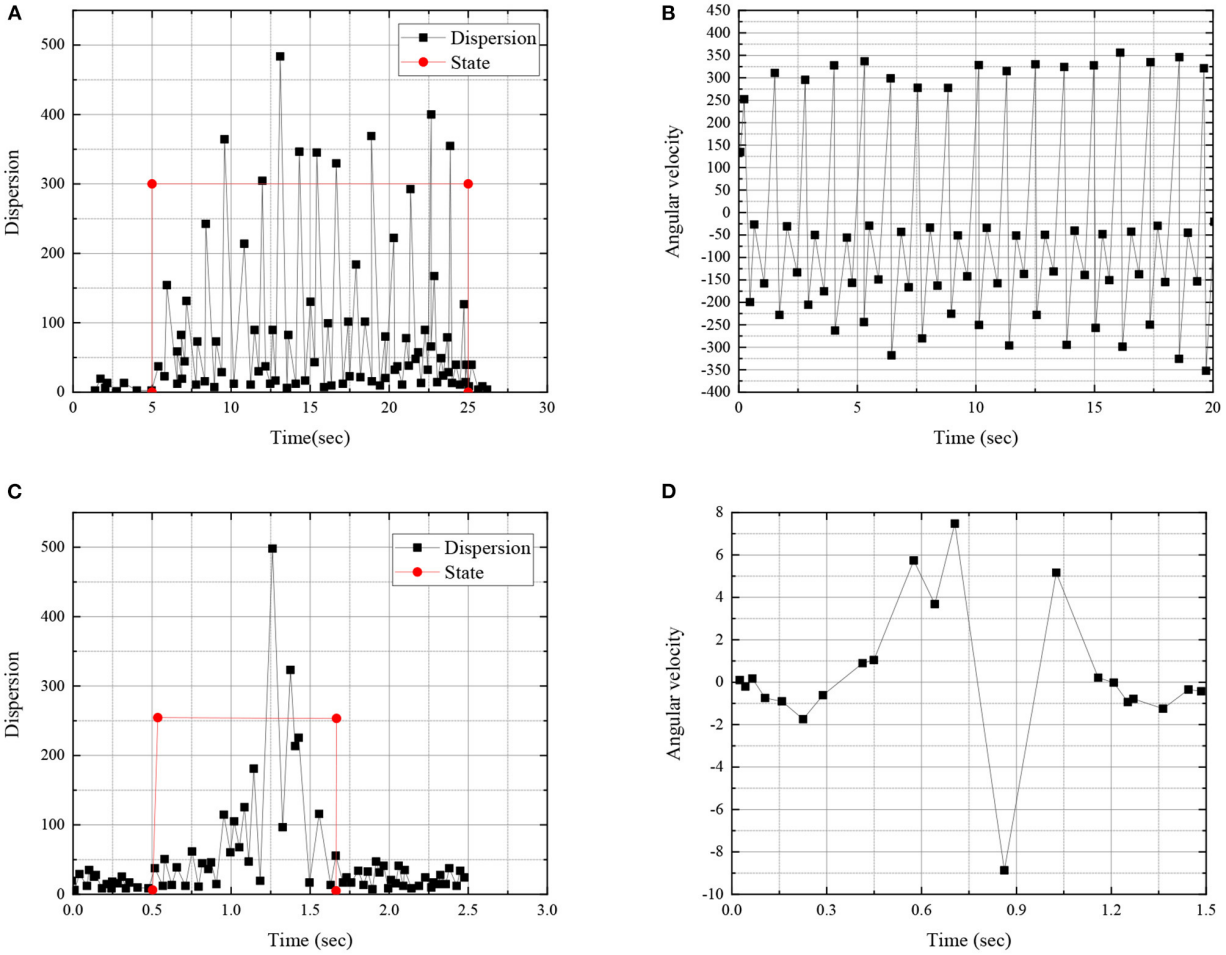
Principle of the data partition method based on discreteness **(A)** angular velocity discreteness and motion state during walking; **(B)** angular velocity of legs during walking; **(C)** angular velocity dispersion and motion state during passing; and **(D)** arm angular velocity during passing.

Through the principle of data division of discreteness, the skill movements of athletes during basketball training are detected, and Figure 7 displays the collected data.

**Figure 8:**
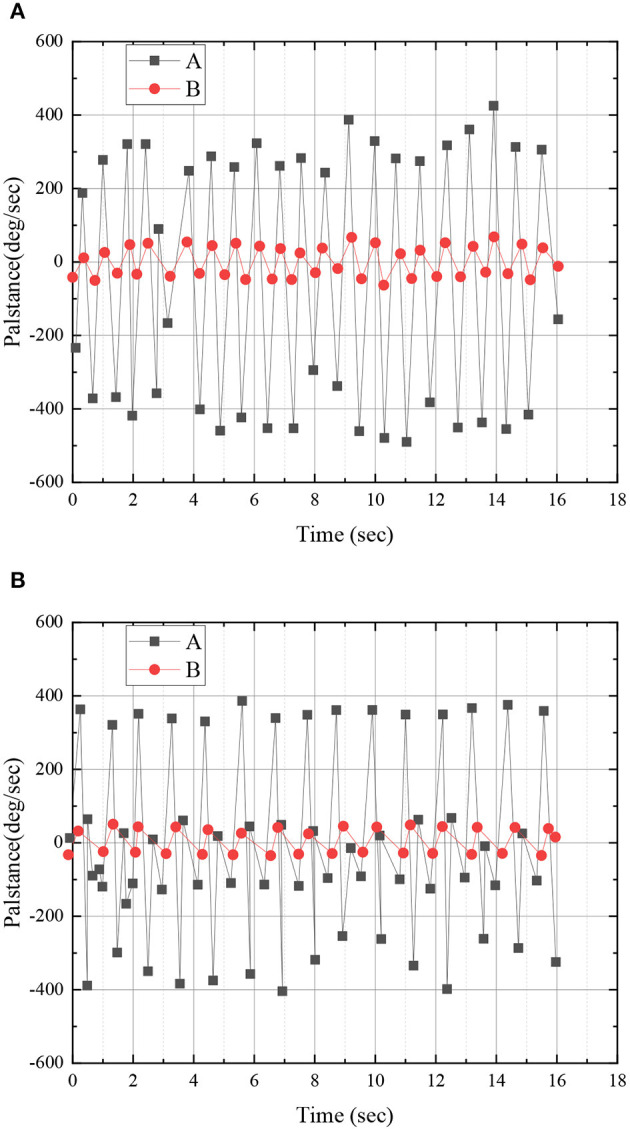
Comparison of the angles and angular velocities of basketball players forearm dribbling and calf walking: **(A)** forearm and **(B)** calf. (A: forearm/calf angular momentum while dribbling and B: forearm/calf angle while dribbling).

Figure 7 shows that, by dividing the action states of basketball players in the training process, it is possible to extract the action data of basketball players in the training process. Figure 8 shows the relative angular velocity and angle analysis between the forearm and calf during walking dribbling.

According to the experimental data in Figure 8, the results indicate that the angular velocity signals detected by basketball players when dribbling on the forearm and walking on the calf have significant noise signals, which lead to the unsmooth signal data curve. However, the angle signal curves detected by basketball players are relatively smooth when dribbling on their arms and walking on their calves. Therefore, the conclusion suggests that the angle can be taken as the division of unit actions, reducing the complexity.

### Basketball Posture Recognition Test

The test of basketball gesture recognition is to verify the effect of basketball gesture recognition. The scenes are set of various basketball actions. The physical information data are obtained from the different detectors in varying basketball actions. Experiment, a large amount of data are collected. The corresponding experimental scene is also tested. The collected data are processed according to the described data processing and data division methods. The feature vector set describing the specific action is obtained. Thus, the sample set is constructed. Finally, the sample set is sent to the classifier. Here, the realization of the classifier uses the existing Weka platform to compare and analyze the performance of several classifiers.

The experiment will organize the walking, running, and jumping motion data of eight male testers without the ball to collect basketball motion data. When holding the ball, the motion data of stationary dribble, walking dribbling, running dribble, shooting, passing, and catching are collected separately. Each action is repeated for 50 times. There are 5,000 samples in total. When holding the ball, the upper body movements included stationary dribble, walking dribble, running dribble, shooting, passing, and catching, a total of 2,400 times. There are 2,600 lower body movements, including walking without the ball, running, jumping, walking, dribbling running, dribbling, and shooting. Each tester completed the required actions as required during the sampling process and monitored the recorded amount of activity. Table 1 presents the statistical results of the samples collected by each tester during the data collection process. Table 2 shows the statistics of different movements of the upper and lower extremities.

**Table 1:** The number of samples collected by each tester's thinking data.

**Behavior**	**Number of actions**
Walk	50
Running	50
Jump	50
Stationary dribble	50
Walking dribble	50
Running dribble	50
Shot	50
Pass	50
Catch the ball	50

**Table 2:** The number of different movements of the upper and lower limbs.

**Upper and lower limbs**	**Behavior**	**Number of actions**
Arm movements	Stationary dribble	400
	Walking dribble	400
	Running dribble	400
	Shot	400
	Pass	400
	Catch the ball	400
Leg movements	Walk	400
	Running	400
	Jump	400
	Shot	400
	Running dribble	550
	Walk dribble	450

**Figure 9:**
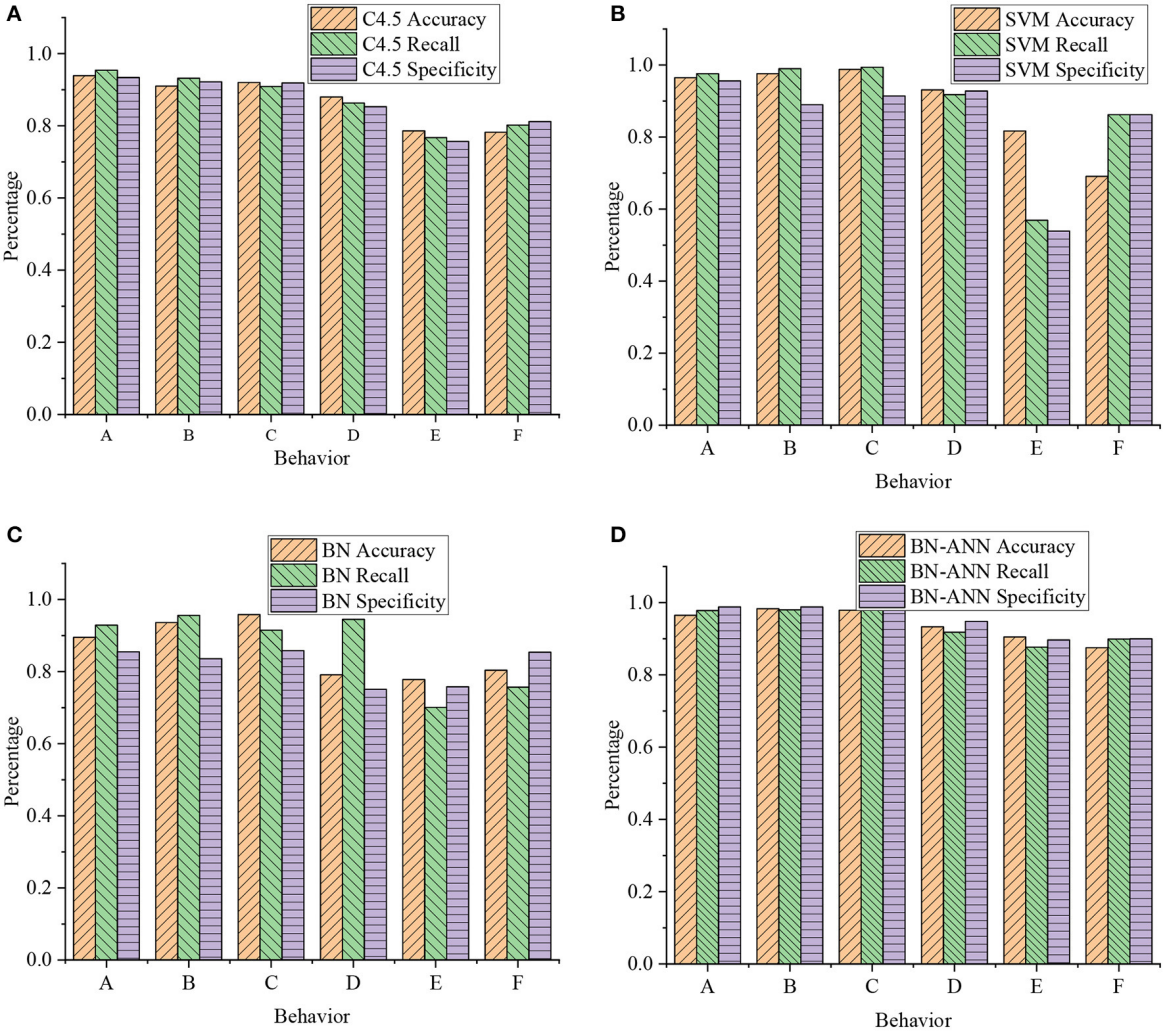
Classification results of upper limb movements by different classification algorithms (A: catching; B: passing; C: shooting; D: dribbling on foot; E: dripping while standing; F: running dribbling). **(A)** accuracy and recall of C4.5; **(B)** accuracy and recall of SVM; **(C)** accuracy and recall of BN; and **(D)** accuracy and recall of back propagation–artificial neural network (BP-ANN)].

**Figure 10:**
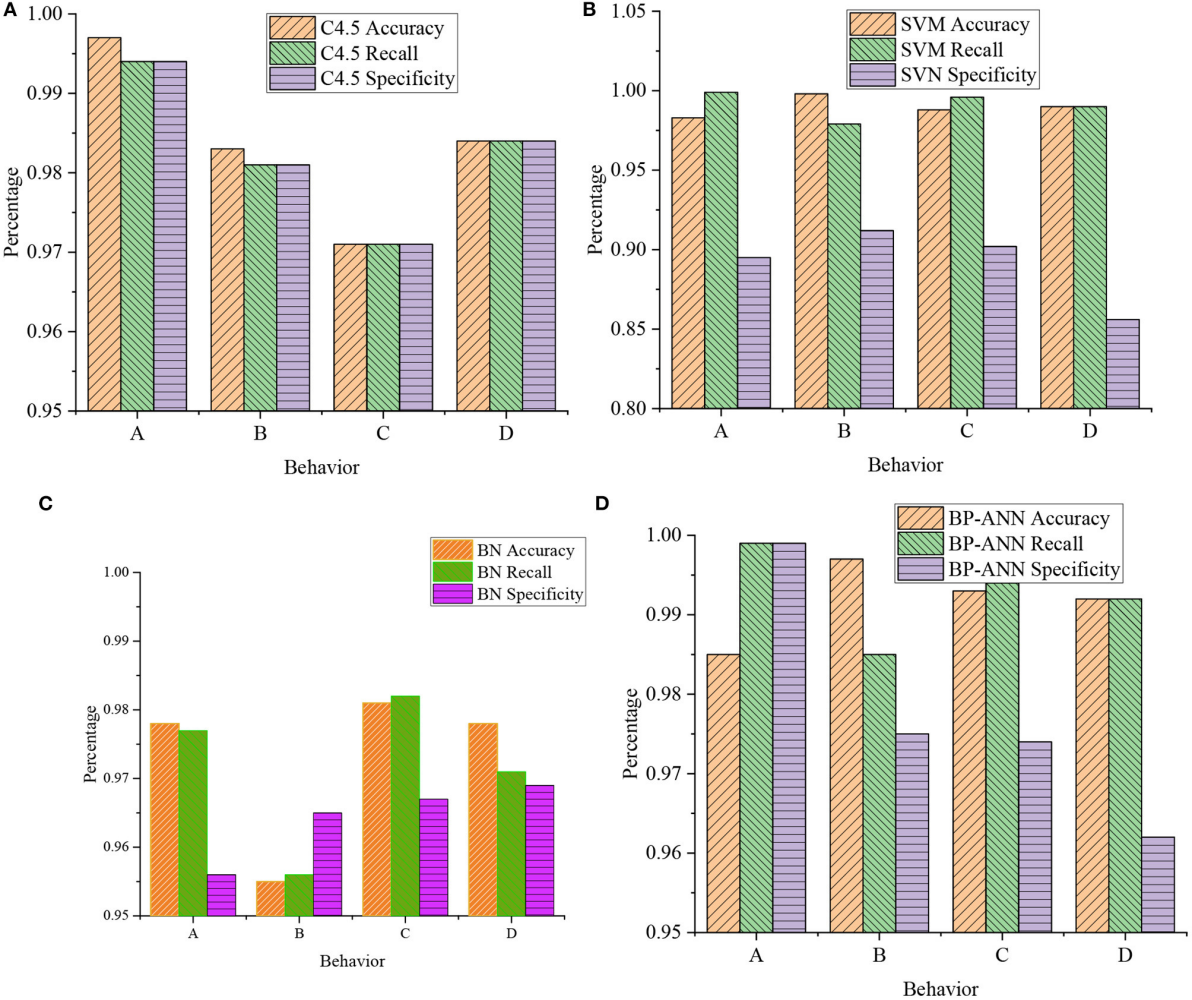
Classification results of lower limb movements in basketball training by different classification algorithms (A: jumping; B: running; C: on foot; D: average level). **(A)** Accuracy and recall of C4.5; **(B)** accuracy and recall of SVM; **(C)** accuracy and recall of BN; and **(D)** accuracy and recall of BP-ANN.

Basketball action is mainly an overall movement completed by the coordinated movement of the upper and lower limbs of an athlete. Therefore, when basketball moves are recognized, the upper and lower limb movements are discussed separately. In the data collection process, sensor nodes are placed in different positions of the body, and the data on upper and lower limb movements are collected and discussed, respectively. For upper and lower limb movements, classifiers are constructed separately for recognition. The moves made by the athletes are determined through the combination of upper and lower limb movements. The classification characteristics of different classifiers are analyzed. The classification performance of different classifiers for basketball gesture recognition is compared. A corresponding classification algorithm is constructed for training the motion data of additional limbs. In Figures 9, 10, the whole experimental process is implemented on the Weka platform, and the recognition effect is analyzed from the two aspects of precision and recall. A 10-fold cross-validation method is used.

Figures 9, 10 are obtained through the data analysis of the body movements, as presented in Tables 1, 2. The recognition effect of the back propagation (BP) ANN is better for the action classification of different limbs. Among them, the average accuracy rate of upper limb movements reached 93.2%. The average recall rate reached 93.2%. The average accuracy of lower body movements reached 99.2%. The average recall rate reached 99.2%. For the four recognition algorithms, the average accuracy of lower extremity movements (jumping, running, and walking) ranged from 97 to 99.2%. The average accuracy of upper body movements ranged from 84.9 to 93.2%. The recognition accuracy of upper body movements (catching, passing, and shooting) is relatively low. This is because the upper limb movement states of *in situ* dribbling, walking dribbling, and running dribbling are all dribbling states. The three dribbling characteristics are similar and difficult to distinguish. Meanwhile, the upper body movements of dribbling in place, walking, and running are considered as a movement state. The average recognition rate is up to 99%, and the average recall rate is up to 99%.

### Analysis of Experimental Results of Different Classification Algorithms

**Figure 11:**
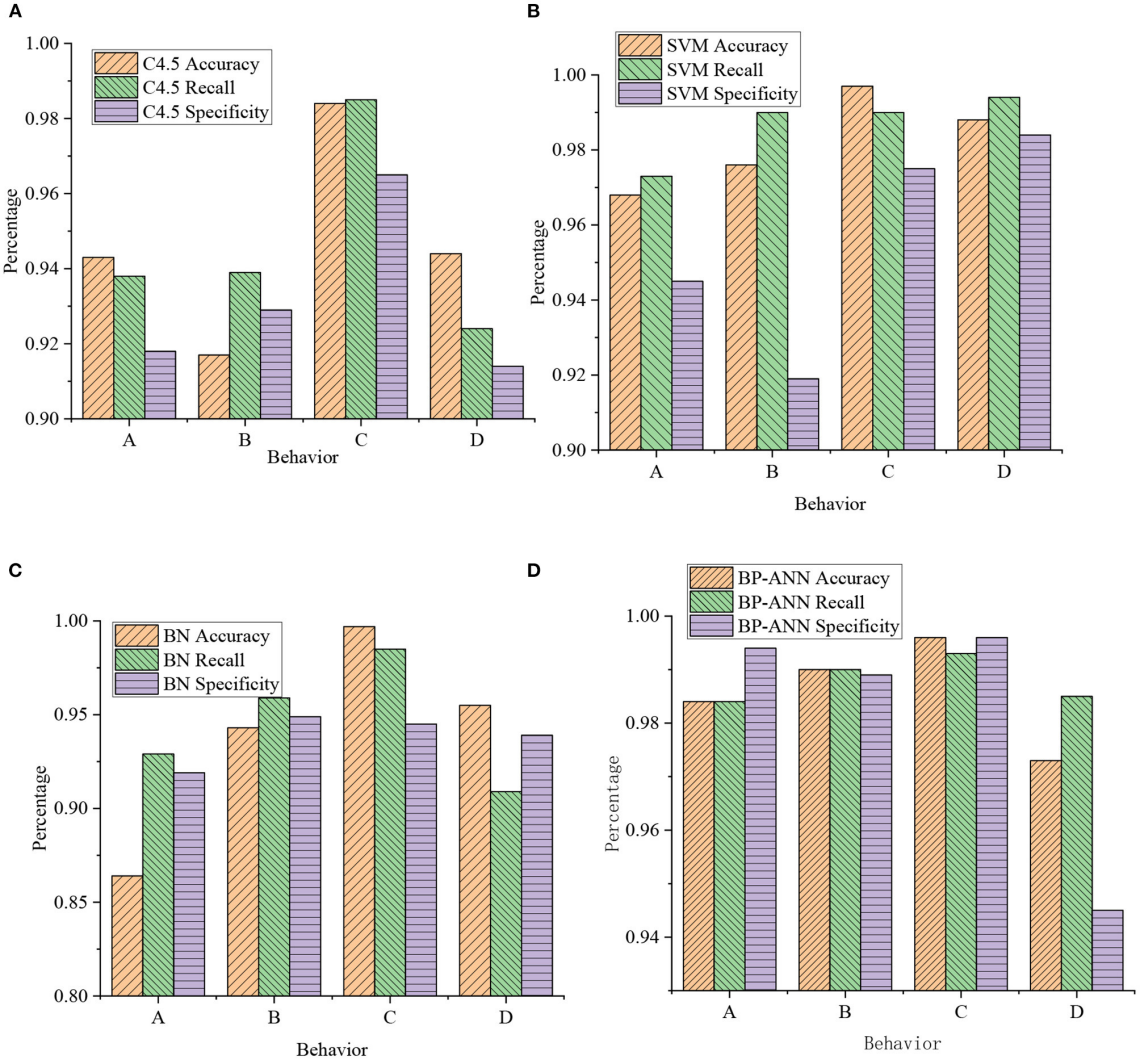
Classification of upper limb movements by the four classification algorithms: **(A)** Accuracy and recall of C4.5; **(B)** accuracy and recall of SVM; **(C)** accuracy and recall of BN; and **(D)** accuracy and recall of BP-ANN (A: catching; B: passing; C: dribbling; D: shooting).

Figure 11 shows the classification results of athletes' upper limb movements in basketball training after the four classification algorithms are merged.

Figure 11 shows the classifier established by the BP ANN algorithm, which can efficiently identify the basketball movements of the ball, passing, dribbling, and shooting. In detecting upper and lower limb movements, the recognition rate exceeds 95%. The average accuracy of the four actions is close to 98.95%. Meanwhile, among the four algorithms, the BP ANN algorithm can more accurately identify the technical movements of the upper and lower limbs.

## Conclusion

The angular velocity signal detected by the basketball player's forearm dribbling and calf walking has a significant noise signal. The drawn signal data curve is not smooth enough. The angle signal curve detected by the basketball player's forearm dribbling and calf walking is relatively smooth. Therefore, the division of angles as unit actions can reduce the complexity. Meanwhile, the BP ANN algorithm showed the best action recognition effect. In the detection of upper limb movements of athletes in basketball training, the average accuracy is close to 93.3%. The average recall is immediate to 93.3%. In detecting lower limb movements of basketball-trained athletes, the average accuracy rate is close to 99.4%. The average recall is close to 99.4%. In detecting upper and lower limb movements, the BP ANN algorithm is used to build a classifier: the recognition method can efficiently identify basketball movements of ball, passing, dribbling, and shooting with a recognition rate of over 95%. The average accuracy of the four actions is close to 98.95%. This can accurately collect the sports parameters of the athletes in real-time, analyze and identify the sports postures of the athletes, and build a training effect evaluation model. The coaches make reasonable adjustments to the training program and scientifically evaluate the training quality. This is of great significance to improving athletes' competitive ability and coaches' decision-making ability.

However, this study also has certain limitations. The technical movements of basketball training are not rich enough, resulting in preliminary results. In the future, the basketball teaching assistant robot should combine the trainee's different action designs and tactical combinations by meeting the trainee's needs efficiently and purposefully. The robot will be equipped with various peripheral sensors, infrared (IR) sensors, visual sensors, etc., to monitor the target position in real-time and make corresponding defenses and counterattacks. These problems will be further improved to ensure the routine implementation of various assumptions and meet the needs of college physical education teaching basketball tactics.

## Data Availability Statement

The original contributions presented in the study are included in the article/supplementary material, further inquiries can be directed to the corresponding author.

## Author Contributions

All authors listed have made a substantial, direct, and intellectual contribution to the work and approved it for publication.

## Conflict of Interest

The authors declare that the research was conducted in the absence of any commercial or financial relationships that could be construed as a potential conflict of interest.

## Publisher's Note

All claims expressed in this article are solely those of the authors and do not necessarily represent those of their affiliated organizations, or those of the publisher, the editors and the reviewers. Any product that may be evaluated in this article, or claim that may be made by its manufacturer, is not guaranteed or endorsed by the publisher.
